# The Cephalopod Large Brain Enigma: Are Conserved Mechanisms of Stem Cell Expansion the Key?

**DOI:** 10.3389/fphys.2018.01160

**Published:** 2018-08-21

**Authors:** Astrid Deryckere, Eve Seuntjens

**Affiliations:** Laboratory of Developmental Neurobiology, Department of Biology, KU Leuven, Leuven, Belgium

**Keywords:** stem cell, neurogenesis, cephalopod, brain development, invertebrate neuron

## Abstract

Within the clade of mollusks, cephalopods have developed an unusually large and complex nervous system. The increased complexity of the cephalopod centralized “brain” parallels an amazing amount of complex behaviors that culminate in one order, the octopods. The mechanisms that enable evolution of expanded brains in invertebrates remain enigmatic. While expression mapping of known molecular pathways demonstrated the conservation of major neurogenesis pathways and revealed neurogenic territories, it did not explain why cephalopods could massively increase their brain size compared to other mollusks. Such an increase is reminiscent of the expansion of the cerebral cortex in mammalians, which have enlarged their number and variety of neurogenic stem cells. We hypothesize that similar mechanisms might be at play in cephalopods and that focusing on the stem cell biology of cephalopod neurogenesis and genetic innovations might be smarter strategies to uncover the mechanism that has driven cephalopod brain expansion.

## The Complex Brain of Cephalopods

Among mollusks and even among all invertebrates, cephalopods have a large and complex brain that is highly centralized (Nixon and Young, 2003). The brain encircles the esophagus and is divided into 25 major lobes further subdivided in 37 or 38 lobes in octopods and decapods, respectively. These lobes control different functionalities, including motor function, feeding and color change, but also sensory information processing and higher cognitive functioning (Young, 1963, 1971; Budelmann, 1995; Nixon and Young, 2003). The adult cephalopod brain has a typically invertebrate ganglia-like structure with densely packed neural cell bodies lying in the outer, perikaryal layer and branched processes and synapses centered in the neuropil (Matheson, 2002; Richter et al., 2010). It however seems that the cephalopod brain has a cordal origin, meaning that the initially formed cluster of neurons is longitudinally stretched rather than densely packed. This cordal organization is similar to the more primitive aculiferans like the chiton instead of a ganglionic origin shared by conchifera such as gastropods and bivalves (Richter et al., 2010; Shigeno et al., 2015). In this simplistic system of cords, neurons are allocated in rope-like territories in the neurectoderm, spanning the midline of the early embryo. The brain then develops further by increasing the size of these cordal territories by proliferation and migration of neuroblasts and by global transitions to centralize the cords (Yamamoto et al., 2003; Richter et al., 2010; Shigeno et al., 2015). Along with ensheathing neuron fibers with myelin to increase conduction velocity, which is widespread in vertebrates but also invertebrates (Boullerne, 2016), this centralization allows faster information processing leading to more complex behavior (Budelmann, 1995).

Similar to a proposed scenario of nervous system evolution (Arendt et al., 2016), cephalopods might have adopted a simple neural organization (nerve net) and expanded its size (to cord and brain lobe) by enhancing neurogenesis. It is tempting to speculate that an increase in neuronal number would allow complex behaviors and enhanced cognitive capacity. The common octopus for example has the largest and most complex brain of all cephalopods, allowing amazing problem solving capacity (Young, 1971; Fiorito et al., 1990; Nixon and Young, 2003). Its nervous system accounts for about 500 million nerve cells (Young, 1963; Budelmann, 1995) which is seven times more compared to the mouse brain (Herculano-Houzel et al., 2006) and comparable to the marmoset, a small primate (Herculano-Houzel et al., 2007).

Also in vertebrate evolution, increased learning and memory is paralleled by a massive expansion of the cerebral cortex (Abdel-Mannan et al., 2008). What remains puzzling is how certain cephalopods such as cuttlefish, squid and in particular octopus were able to immensely increase their neuronal numbers to 100s of millions, whereas other mollusks (e.g., *Aplysia*: 10,000 neurons) or invertebrates (e.g., *Drosophila*: 135,000 neurons) did not. In this Perspective, we discuss the potential mechanisms that could lead to increased neuronal production in cephalopods from an evolutionary viewpoint and suggest routes for future investigation.

## Conserved Genetic Pathways for Neurogenesis: What Do We Learn?

Several studies on the fruit fly *Drosophila melanogaster*, the annelid *Platynereis dumerilii* and the mouse indicate that divergent species have chosen a similar molecular blueprint to establish their central nervous system (CNS) (Martín-durán et al., 2018). Transcription factors and secreted morphogens that determine the anterior–posterior (Otx-Pax-Hox) as well as dorso-ventral (BMP-Msx-Nkx) patterning of the CNS have been evolutionary conserved and ensure the organized development and position of the CNS in invertebrate and vertebrate species (Hirth, 2010). For example, signaling molecules and transcription factors such as *Nkx2.1*/*Nkx2.2, Pax6*, and *Otx2* are expressed in a comparable pattern along the anterior–posterior and dorsoventral axes in the neurectoderm of *D. melanogaster* and *P. dumerilii* and the dorsal neural plate of vertebrates (Holland et al., 2013; Martín-durán et al., 2018).

In cephalopods, the expression of these and other general neuroectodermal patterning transcription factors has also been conserved (Navet et al., 2014; Wollesen et al., 2014). *Pax2/5/8* expression in the CNS of the pygmy squid *Idiosepies notoides* demarcates roughly comparable anterior–posterior patterning as *Drosophila* and mouse, positioning the structures that are responsible for higher cognitive functioning and signal integration, such as the superior frontal and the vertical lobe, at the most anterior end (Wollesen et al., 2015). A similar study in *Sepia officinalis* shows that the mediolateral patterning of the CNS marked by *Nkx2.1, Pax2-8, Gsx*, and *Msx* seems grossly conserved, although the orientation has been reversed (Buresi et al., 2016). Furthermore, the collinear anterior to posterior expression pattern of Hox genes is preserved in the CNS of the squid *Euprymna scolopes* (Lee et al., 2003). Shh, a morphogen and the transcription factor Pax6 have been extensively studied in vertebrates and *Drosophila* where they steer eye formation and are involved in nervous system development by specifying dorsoventral identity (Echelard et al., 1993; Halder et al., 1995; Ericson et al., 1997). In the cuttlefish *S. officinalis* and squids *Loligo opalescens* and *E. scolopes, Pax6* expression is found in the developing eyes, suckers of the arms and in the optic lobes (Tomarev et al., 1997; Hartmann et al., 2003; Navet et al., 2009). In *S. officinalis*, expression was also observed in visceral and cerebral ganglia, but unlike in vertebrates, *Pax6* expression is not restricted to the dorsal area of the CNS and *Shh* is constrained to tissues surrounding the optic area (Navet et al., 2009, 2014).

Taken together, several conserved transcription factors and morphogens are expressed in developing cephalopod brains, in patterns that remain grossly similar to other invertebrates. However, signaling factors such as Wnt, TGF-β, Hedgehog, FGF, and Notch as well as transcription factors such as SoxB and proneural basic helix-loop-helix proteins have been found to be implicated in neurogenesis and the formation of neural networks in *Nematostella vectensis*, a cnidarian without a centralized and expanded brain (Rentzsch et al., 2017). It therefore, remains questionable whether the presence of conserved neurogenic factors in itself will be key to reveal the mechanism behind the remarkable expansion of neural tissue in coleoid cephalopods. Indeed, not only the presence of such factors is important, their function needs to be preserved as well. The latter is not always the case: bivalves and gastropods adopted the expression of posterior markers of brain development such as *Gbx* to develop a shell: a different, typical mollusk feature that is absent in coleoid cephalopods (Wollesen et al., 2017). This finding indicates we might not discover the (molecular) mechanism driving neurogenic expansion by examining merely the presence of conserved molecular pathways. In addition, it will be required to investigate functional conservation. Furthermore, as will be explained below, the neurogenic process itself could be studied more from a cell biological viewpoint, especially in species that evolved out of the ordinary.

## Mechanisms to Increase Neuronal Cell Number

**FIGURE 1:**
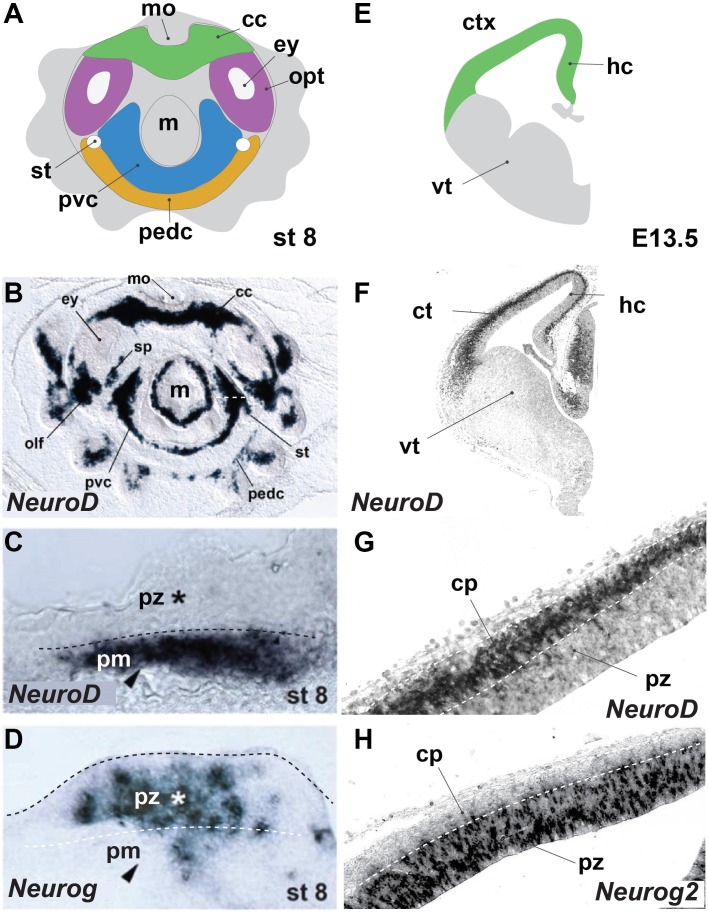
*Octopus bimaculoides* and mouse neurogenesis occurs in similarly laminated neuroectoderm. **(A)** Schematic top–down overview of the neurogenic territories in the stage 8 *Octopus bimaculoides* embryo. All color-marked areas are neurogenic, cord-like regions. **(B)** Whole-mount *in situ* hybridization for NEUROD, a marker of young post-mitotic neurons. **(C,D)** Higher magnification of the neurogenic area (white dashed line in **B**) demarcating a laminated structure with post-mitotic neurons (pm, arrowhead, marked by *NEUROD*, **C**) separated from progenitors (pz, star, marked by *NEUROG*, **D**) (dashed line). **(E)** Schematic view of a coronal section through the mouse telencephalon at E13.5, demarcating the ventral telencephalon (vt, gray) and dorsally placed cortex (ctx) and hippocampal (hc) areas (green). **(F)** *In situ* hybridization of *Neurod*, a post-mitotic neuron proneural transcription factor. **(G,H)** Higher magnification of the cortical laminated structure (dashed lines), with a progenitor zone (pz, marked by *Neurog2*, **H**) lining the ventricle and a post-mitotic cortical plate (cp, marked by *NeuroD*, **G**). **(B–D)** Adapted from Shigeno et al. (2015). cc, cerebral cord; cp, cortical plate; ctx, cerebral cortex; ey, eye; hc, hippocampus; m, mantle; mo, mouth; olf, olfactory organ; opt, optic lobe; pedc, pedal cord; pvc, palliovisceral cord; pz, progenitor zone; sp, subpedunculate tissue; st, statocyst; vt, ventral telencephalon; ve, ventricle.

**FIGURE 2:**
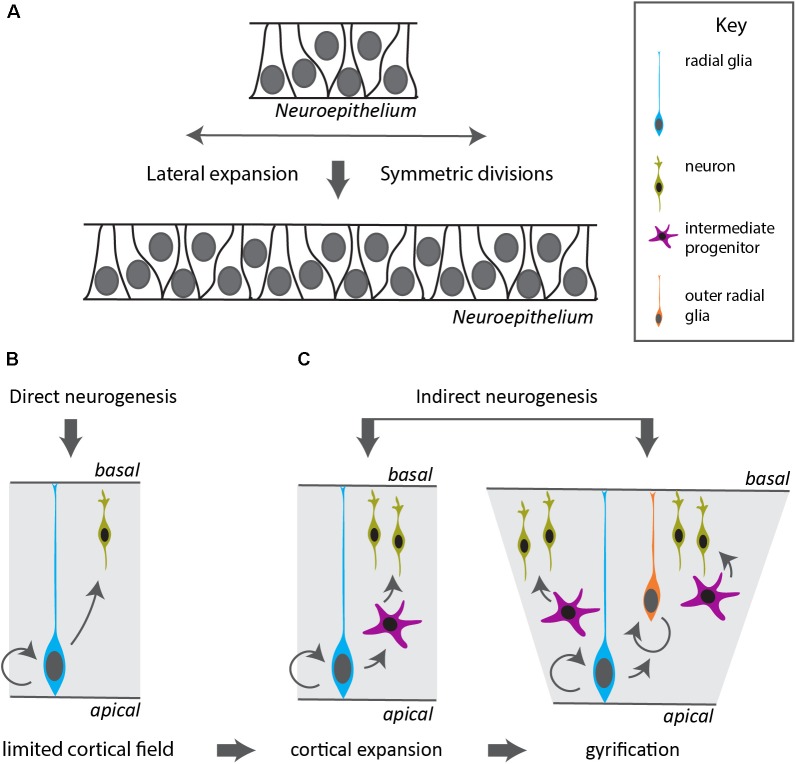
Modes of neurogenesis in the vertebrate cerebral cortex. **(A)** Before the onset and during early stages of neurogenesis, the neuroepithelium divides symmetrically to expand in a lateral fashion, increasing the neurogenic domain. **(B)** In vertebrates with a small cortical field, the radial glia divide asymmetrically to generate neurons in a direct manner. **(C)** Indirect neurogenesis generates intermediate progenitors that divide symmetrically resulting in increased neuronal output and expansion of the cerebral cortex. The appearance of a duplication of the radial glia layer in outer radial glia allows further radial and lateral expansion and gyrification of the cortex.

Examples of neural expansion in terms of cell number can be found most prominently in vertebrates, in the most anterior part of their CNS, the telencephalon. Shortly after neurulation, the neural tube extends in a lateral fashion by symmetric divisions of the neuroepithelial precursors. This leads to an expansion of the neurogenic domain and happens before the generation of neurons (Fish et al., 2008). Such a lateral expansion goes beyond typical neuroectodermal invaginations observed in other deuterostomes and ecdysozoans (Hartenstein and Stollewerk, 2015). A broad neurogenic domain is also apparent during cephalopod development. The cephalopod brain emerges from four pairs of ectodermal placodes in the equatorial zone of the embryo, that develop into rope-like territories (Yamamoto et al., 2003). At the onset of organogenesis, these neurogenic precursor regions occupy a major part of the cephalopod embryonic ectoderm as was shown by expression of *SoxB1* (Buresi et al., 2016) and represented by color-marked areas in **Figure 1A**. At the same time, there is evidence of early post-mitotic neurons expressing synaptotagmin or *NeuroD* (**Figure 1B**; Shigeno et al., 2015; Buresi et al., 2016). Interestingly, the cephalopod neurogenic territory is layered, and post-mitotic neurons (pm) form a distinctive band toward the inside (**Figure 1C**), whereas progenitors form a distinct sheet on top (**Figure 1D**; Shigeno et al., 2015). A similar division occurs in mammalian cortical neurogenesis (**Figure 1E**), where post-mitotic neurons (marked by *NeuroD*, **Figures 1F,G**) migrate radially outwards to form the cortical plate, leaving the progenitors (marked by Neurogenin2, **Figure 1H**) as an apical layer surrounding the ventricle (**Supplementary Material**). Also in the teleost fish telencephalon, post-mitotic neurons migrate radially inwards and progenitors remain as a distinctive layer at the outside apical border (Abdel-Mannan et al., 2008; Furlan et al., 2017). Neurogenesis in the vertebrate cerebral cortex is marked by a switch from symmetric to asymmetric divisions, in which the neurogenic stem cell (also known as radial glia, blue cell in **Figure 2**) self-renews and generates a daughter cell that either becomes post-mitotic (direct neurogenesis), or an intermediate precursor (indirect neurogenesis) (**Figure 2**; Paridaen and Huttner, 2014). These intermediate progenitors (multipolar pink cells in **Figure 2**) divide a few times to generate the bulk of the post-mitotic neurons (labeled green in **Figure 2**), that actively migrate out of the progenitor domain. In vertebrates, the increasing ratio between indirect and direct neurogenesis determines the radial expansion of the cortex seen over evolution (Florio and Huttner, 2014; Taverna et al., 2014). Besides controlling the decision between proliferative symmetric over neurogenic asymmetric divisions, and between direct versus indirect neurogenesis, diversifying the nature of the intermediate progenitors is a third way particularly managed by mammals (including primates and human) to vastly increase neuronal number. Evidence exists that duplication of the radial glial neurogenic stem cell layer resulting in the formation of basal (or outer) radial glia (orange cells in **Figure 2**) lies at the basis of gyrification of the cerebral cortex (Florio and Huttner, 2014; Fernández et al., 2016). The columnar organization of the amacrine cells around their bundled trunks in octopods we observe in drawings from both of Gray and Young, and the folded structure of the vertical lobe (Gray, 1968; Young, 1971), are reminiscent of the primate cerebral cortex structure (Hubel and Wiesel, 1969). Compared to other invertebrates such as *Drosophila*, that also has different types of neurogenic precursors (Homem and Knoblich, 2012; Hartenstein and Stollewerk, 2015), cephalopods might have increased neuronal output applying vertebrate-like mechanisms (symmetric divisions to laterally expand the neurogenic stem cell field, larger diversity of progenitors to increase indirect neurogenesis and active neuronal migration).

Regulation of the cell cycle is obviously important in the context of neurogenesis. A prolonged period of active cell cycling in neural stem cells would be an additional mechanism to increase neuronal output. In humans, primary microcephaly, which is due to lower cortical cell number and manifests as a reduction in cortical size, is caused by mutations in genes important in mitotic cell division, such as spindle formation and centrosome function (Gilmore and Walsh, 2013). The fact that these mutations primarily affect brain development suggests that factors that control cell cycle will predominantly impact the number of neurons produced in an animal. Assuming that a basic process such as the cell cycle is regulated by similar factors in all bilaterian animals, has very recently been put into question. The planarian *Schmidtea mediterranea* seems to have lost *MAD1, MAD2* and several other genes implicated in the spindle assembly checkpoint (Grohme et al., 2018). These factors have conserved functions from yeast to mammalians, yet seem not that essential to planaria, that have retained mitotic checkpoint function. Amazingly, planarians display whole-body regeneration potential while overproliferation conditions such as cancer have not been reported. Similar gene losses have been described in *Drosophila* that should affect DNA repair, yet this process is not really impacted either (Sekelsky, 2017). Clearly, our knowledge on basic cellular processes such as the cell cycle is far from complete, in particular in cephalopods.

## Adult Neurogenesis

The cephalopod brain continues to grow over the entire lifetime of the animal (Wirz, 1959; Young, 1963; Dickel et al., 1997) whereas particular regions such as the vertical lobe and superior frontal lobe increase in size in response to learning (Dickel et al., 2001). This growth is paralleled -at least in *O. vulgaris*- by a linear increase in DNA content and number of nuclei (Packard and Albergoni, 1970; Giuditta et al., 1971), suggesting that also beyond embryogenesis, neurons are generated. In mammals, adult neurogenesis is steered by neural stem cells in the ventricular-subventricular zone lining the lateral ventricle and in the subgranular zone of the hippocampal dentate gyrus (Zhao et al., 2008; Altman, 2011). Non-mammalian vertebrates like teleosts bear neural progenitors in multiple neurogenic regions. These continuously produce new neurons that migrate and integrate in the mature brain (Kizil et al., 2012). Adult neural stem cells in invertebrates have also extensively been studied (reviewed by Simões and Rhiner, 2017). *D. melanogaster* only shows a low level of adult neurogenesis by a dispersed population of neural progenitors in the optic lobes. These progenitors are mainly quiescent (Fernández-Hernández et al., 2013), but can start local proliferation upon acute tissue damage (Heisenberg et al., 1995; Moreno et al., 2015).

To date, little information exists on adult neurogenesis in cephalopods. Buresi et al. (2013) suggest a prolongation of proliferative capacities of the ganglia in cephalopod hatchlings which implies preservation of quiescent stem cells to allow delayed adult neurogenesis (Baratte and Bonnaud, 2009). Excitotoxic lesion by kainic acid in the vertical lobe of *S. officinalis* induced proliferation as measured by BrdU incorporation (Graindorge et al., 2008) and recently, Di Cosmo et al. (2018) observed active proliferation in the *O. vulgaris* nervous system after *in vitro* administration of BrdU. PCNA levels seem to increase in the vertical and frontal lobes of *O. vulgaris* housed in an enriched environment suggesting active cell division takes place (Bertapelle et al., 2017), however, leaving the reader in the dark on the precise cellular location of the presumptive raise in mitotic activity. Whether these findings reveal a true self-renewing population of stem cells and whether neurons are generated is therefore not yet proven. Measurements of DNA content per nucleus in different adult *O. vulgaris* brain lobes indicated an amount that exceeded the estimated DNA content of diploid cells, suggesting polyploidy in a number of cells (Giuditta et al., 1971). Polyploidy might indicate active cell cycling (tetraploidy during G2 phase). Intriguingly, a recent report showed that during starvation stress, stem cells can be generated from polyploid cells by amitosis in *Drosophila* (Lucchetta and Ohlstein, 2017). This alternative mechanism of cell division that is characterized by nuclear division without spindle formation, has been shown to occur in many species ranging from plants and ciliates to mammals (Miller, 1980; Kuhn et al., 1991; Magelhães et al., 1991; Prescott, 1994) and might be induced by physiological and pathological stressors (Chen and Wan, 1986). Lange (1920) already suggested a role for direct division or amitosis in octopus arm regeneration after amputation. She did not observe infiltrating cells nor mitotic spindles in the blastema-like structure, but instead found several nuclei in different stages of amitotic division (Lange, 1920). Given that such alternative mechanisms to mitosis might exist, and quiescence of adult progenitors might “hide” neurogenesis, more extensive exploration of adult neurogenesis that goes beyond demonstration of mitosis is necessary. An interesting alternative route of neurogenesis was described recently. In crustaceans, the adult pool of neurons is supplied from the hematopoietic system that act as true stem cells to sustain neurogenesis in the adult animal (Benton et al., 2014).

## Genetic Innovations Might Drive Complex Neural System Development

Sequencing of *Octopus bimaculoides* revealed an extremely large genome size [∼2.7 versus ∼1.6 Gb for *Mytilus* (mussel)] (Albertin et al., 2015; Murgarella et al., 2016). Unexpectedly, this increase is not due to simple duplication, but by expansion of a few specific gene families including protocadherins and C_2_H_2_ zinc finger proteins, as well as interleukin-17-like genes, G-protein coupled receptors, sialins and chitinases (Albertin et al., 2015). A similar protocadherin gene expansion has been found in coleoid cephalopods (Liscovitch-Brauer et al., 2017), whereas cadherin expression is enriched in suckers, such as for instance the unique *CDHX* (Wang and Ragsdale, 2017). Protocadherins have predominant functions in the development and maintenance of the nervous system of vertebrates and are highly enriched in neural tissue of *O. bimaculoides*, but are absent in *Drosophila* (Zipursky and Sanes, 2010; Liu et al., 2014; Wang et al., 2014; Albertin et al., 2015). Furthermore, Albertin et al. (2015) identified three copies from the disc large family members in the *O. bimaculoides* genome. Members of this family function in post-synaptic scaffolding and have four copies in the mammalian genome whereas *Drosophila* only has one (Nithianantharajah et al., 2013; Albertin et al., 2015). The independent expansion of these and more genes in both vertebrates and *O. bimaculoides* and their enrichment in neural tissues, suggest a convergent evolution on the molecular basis and might be related to an increasingly complex brain. Having a reference genome at hand, we can now hunt for the innovations in the octopus genetic information that might explain their unique neural expansion.

## Future Perspectives

Cephalopods have developed an expanded and centralized CNS that allows amazing behavior and complex cognition. Studying the onset and precise timing of neurogenesis in relation to the diversity of progenitors and neurons will be fundamental to further map out the molecular mechanisms driving cephalopod neural expansion. Hereto, an extra effort to sequence the genomes of cephalopods is essential.

Developing tools for cell biological analysis such as stem cell or explant cultures would allow analysis of cell cycle parameters and neurogenesis. The general lack of information on stem cells for the whole mollusk phylum including around 85,000 extant species hinders setting up *in vitro* cell cultures (Rosenberg, 2014; Hartenstein and Stollewerk, 2015). Indeed, cell culture has not been very successful in mollusks, and only one cell line (Bge cells) has been established so far, derived from embryonic tissue of the snail *Biomphalaria glabrata*, whereas over 500 cell lines of insects exist (Lynn, 2007; Yoshino et al., 2013). Recently, Maselli et al. cultured adult *O. vulgaris* neurons and showed that successful adhesion and neurite extension is limited to 4 days *in vitro* (Maselli et al., 2018). Besides cell culture, brain slice culture has been successfully used to measure long term potentiation in the adult brain (Hochner et al., 2003). These methods deserve further exploration in the context of neurogenesis as well.

Finally, recent genome data of regenerating animals reveal that our knowledge on the regulation of the cell cycle, and by extension the regulation of neurogenesis, is far from complete (Grohme et al., 2018; Nowoshilow et al., 2018). The careful analysis of cell cycle regulation and the prevalence of potentially alternative mechanisms in cephalopods merits further attention.

Taken together, generation and exploitation of additional genome and transcriptome data will yield more insight into the molecular mechanisms of neural expansion, whereas the establishment of (stem) cell culture methods will boost deeper understanding of cell cycle regulation and neurogenesis. Such studies should go hand-in-hand with *in vivo* analysis of the cell biology of neurogenesis during development and in adult life, to understand how this process contributes to brain expansion and plasticity.

## Ethics Statement

All animal experiments were carried out on euthanized animals in compliance with the most recent European regulations and Belgian law and according to the guidelines of the Animal Care Committee of KU Leuven (ECD No. P153/2012).

## Author Contributions

AD and ES wrote the manuscript and approved the final document. ES performed the experiments and drew the figures.

## Conflict of Interest Statement

The authors declare that the research was conducted in the absence of any commercial or financial relationships that could be construed as a potential conflict of interest.
