# Supplementary material for: The Cephalopod Large Brain Enigma: Are Conserved Mechanisms of Stem Cell Expansion the Key?
Source: Front Physiol. 2018 Aug 21;9:1160. doi: 10.3389/fphys.2018.01160 (PMC6110919; doi:10.3389/fphys.2018.01160)
Supplement: Supplementary file 1 [file Data_Sheet_1.pdf]

Supplementary Material

The cephalopod large brain enigma: are conserved mechanisms of stem cell expansion the key?

Astrid Deryckere[1], Eve Seuntjens[1*]

[1]Laboratory of Developmental Neurobiology, KU Leuven, Department of Biology, Leuven, Belgium

*** Correspondence:**
Corresponding Author
eve.seuntjens@kuleuven.be

1 Supplementary Material: Methods

1.1 Mice

All animal experiments were carried out on euthanized animals in compliance with the most recent European regulations and Belgian law and according to the guidelines of the Animal Care Committee of KU Leuven (ECD n° P153/2012). Mice were maintained in a CD1/Swiss background. The day on which the vaginal plug was found, was considered as embryonic day (E) 0.5.

E13.5 brains were dissected and washed in ice-cold PBS, fixed overnight in 4% paraformaldehyde, followed by progressive alcohol-assisted dehydration and paraffin embedding (Thermo Electron Corporation, Shandon Excelsior ES). 6 µm thick coronal sections of embryonic brains were obtained with a microtome (Microm HM360).

1.2 *In situ* hybridization

Paraffin sections were processed for chromogenic *in situ* hybridization using anti-sense riboprobes labeled with digoxigenin-UTP (Roche) on an automated platform (Ventana Discovery, Ventana Medical Systems, Roche). 150 ng of *in situ* probe was used for each reaction together with Ribomap and Bluemap kits (Ventana Medical Systems, Roche). A detailed procedure including pre-treatment, hybridization temperature, stringency wash and color development are available on request. After this procedure, slides were dehydrated and mounted with Eukitt (Sigma). Sections were photographed using a Leica DMR microscope connected to a Spot camera (Visitron Systems). Plasmids for Ngn2 and NeuroD probes were obtained from Q. Ma (Caltech, US) and J. Lee (U. Colorado, US) respectively.
